# Editorial: Assistance Dogs for People With Disabilities

**DOI:** 10.3389/fvets.2020.00087

**Published:** 2020-02-21

**Authors:** Emily Patterson-Kane, Mariko Yamamoto, Lynette Arnason Hart

**Affiliations:** ^1^Independent Researcher, Rolling Meadows, IL, United States; ^2^Department of Animal Sciences, Teikyo University of Science, Yamanashi, Japan; ^3^Department of Population Health and Reproduction, School of Veterinary Medicine, University of California, Davis, Davis, CA, United States

**Keywords:** dog training, emotional support animals, psychiatric service dogs, service dogs, veterinary care

The use of assistance dogs has a long and honorable history. Guide dogs have been held in high regard since the 1930s and since the 1980s, assistance dogs have rapidly expanded to fill new roles, particularly in the U.S. (1). Alongside these burgeoning possibilities for canine assistance, the social and regulatory environment for these dogs has become increasingly complex and some areas of confusion and social conflict have emerged (2). The nomenclature used in describing these dogs adds confusion: at the worldwide agency Assistance Dogs International (ADI), the inclusive term used is *assistance dogs* for guide, hearing and service (all other assisting roles, including for autism or psychiatric disabilities) dogs (3). In contrast, the U.S. American Disabilities Act uses the inclusive term *service dogs* (4).

Veterinarians and social scientists have special responsibilities to work together to support people with disabilities and their assistance dogs. This requires a seamless integration of animal and human medicine that includes a full range of service providers. This “One Health” world is more often aspirational than actual, and scientists and professionals are critical to bridging this gap.

Many highly capable agencies support people with disabilities, provide and support service animals, and advocate for them. However, it is researchers, veterinarians, and human health providers that make the connections between domains that are needed to allow the assistance-dog handlers to carry out their everyday activities with the ease and access that is their right.

This Research Topic aims to showcase some of the work being done to find a constructive way forward, expanding the effective and responsible employment of assistance dogs while managing the associated risks and conflicts. This includes supporting research into efficacy and best practices, promoting wider access to and for assistance dogs, and developing the support systems for handlers and their dogs.

## Improving Placements and Access to Assistance Dogs

It has become almost a cliché to say that more research is needed, but this is an area where research is critically important as the use of assistance dogs grows, vulnerable individuals are affected, and public confusion is rife. The article by Fausak discusses how to conduct literature searches for existing research in this multidisciplinary domain. From this base it becomes apparent that there are many areas where further research would help unblock the paths to progress.

One important goal is to objectively determine the benefits of assistance dogs in relation to different populations of handlers. Wilson et al. demonstrate a method for assessing diabetic alert dogs and understanding the factors that contribute to their levels of performance. Bray et al. show how dogs' performance can be assessed and predicted at a programmatic level to improve the success of assistance dog training and placement. Yamamoto and Hart describe some of the challenges for the growing number of people who are self-training their assistance dogs. Effective outreach is needed for handlers who choose to self-train their dogs so they can benefit from best practices and objective data about their dog's performance.

As these methods to validate and refine assistance dog training and placement evolve, it becomes apparent that there are also geographic obstacles to access. Walther et al. show how large areas within North America and Canada are not easily served by providers of appropriately trained service dogs. Also concerned with handlers' access to well-trained dogs, Takayanagi and Yamamoto describe strategies for increasing the availability of assistance dogs to people with disabilities in Japan.

The social mandate for assistance dogs is sometimes tenuous; media reports continue to reflect confusion about rights of access, and report handlers being denied access to public venues. In the United States, both pets and emotional support animals have important roles. In addition, the role of the assistance animal needs to be appreciated as distinct in terms of the dog's function and the handler's rights of access.

The role of psychiatric service dogs continues to be under-appreciated and subject to unjustified regulatory restriction. Lloyd et al. demonstrate the important roles of psychiatric service dogs for people with mental health disorders and LaFollette et al. examine the effects of different training methods with service dogs assisting veterans with PTSD.

## Optimizing the Working Partnership of Handler and Dog

The special bond between a handler and dog must be celebrated and supported with a planned awareness that this relationship will ultimately come to an end. Ongoing research is helping us understand how to navigate these difficult transitions in a way that supports handlers continuing to appreciate the support of the animals in appropriate and beneficial roles.

Veterinarians and paraprofessionals have an important role in providing care to assistance dogs throughout their lives. This includes veterinarians developing the ability to provide routine preventative and wellness care that addresses special needs of clients as discussed by Grigg and Hart. The veterinary team is particularly vital when managing a dog's retirement or end-of-life care as addressed in papers by Ng and Fine, and Villalobos.

Members of medical and veterinary medical professions have an important duty to facilitate the role of assistance dogs in the lives of people with disabilities. This includes both a basic level of care that should be expected of the medical and veterinary practitioners involved, and the work of specialists who assist in identifying, developing, and providing assistance dogs to handlers. This duty has many difficult aspects, including protecting the welfare of animals and people, and communicating calmly and consistently with the general public.

## Conclusion

As the benefits of assistance dogs in relation to a spectrum of disabilities become more apparent, sound research for assessing the efficacy and determining best practices will become ever more important to protect vulnerable individuals and the interests of the community. It is vital that responsible members of the medical and veterinary professions, and evidence-based programs, retain the initiative in diagnosing the need for assistance dogs, as well as training, placing, and monitoring their use. In this way assistance dogs will continue to develop as a vital method for accommodating the needs of people with disabilities as they fully participate in modern society.

## Author Contributions

EP-K drafted the initial document. MY and LH added edits and all agreed on the final version.

### Conflict of Interest

The authors declare that the research was conducted in the absence of any commercial or financial relationships that could be construed as a potential conflict of interest. The handling Editor declared a shared affiliation, though no other collaboration, with one of the authors LH.
